# Barriers and Facilitators of International Health Care Students’ Well-Being in Higher Education: Protocol for a Systematic Integrative Review

**DOI:** 10.2196/59927

**Published:** 2024-12-11

**Authors:** Yao Xie, Kayode Philip Fadahunsi, Paul Flynn, Simon Taylor-Robinson, Joseph Gallagher, Walter Cullen, John O'Donoghue

**Affiliations:** 1 School of Medicine University College Dublin Dublin Ireland; 2 School of Public Health Imperial College London London United Kingdom; 3 School of Education University of Galway Galway Ireland; 4 Department of Surgery and Cancer Imperial College London London United Kingdom; 5 Business Information Systems Malawi eHealth Research Centre University College Cork Cork Ireland

**Keywords:** integrative review, higher education, international students, educational migrants, barriers, facilitators, well-being, mixed methods synthesis, health care students, health care education

## Abstract

**Background:**

International health care students encounter unique hurdles as they pursue education in foreign countries. These challenges, stemming from adjustment to new cultural environments and stressful academic programs, significantly impact their well-being. Understanding the barriers and facilitators experienced by international health care students is crucial for ensuring their successful integration into academic and professional spheres. Most existing reviews focus on specific populations or disciplines, thus limiting their generalizability.

**Objective:**

This systematic integrative review aims to provide a comprehensive understanding of barriers and facilitators of international health care students’ well-being in higher education.

**Methods:**

The protocol follows the Joanna Briggs Institute’s guidance for a mixed methods systematic review. The main information sources will include PubMed, Scopus, Web of Science, and EBSCOhost, supplemented with manual reference search and citation tracking using Google Scholar. The study selection will be done independently by 2 reviewers based on predetermined eligibility criteria. The study population will consist of international higher education students enrolled in human health–related disciplines including medicine, pharmacy, nursing, and allied health care fields. Qualitative and quantitative data relating to barriers and facilitators of international health care students’ well-being will be extracted using a customized data extraction template in Covidence review management software. Quantitative data will be “qualitized” and integrated with qualitative data using a convergent integrated approach, as described in the Joanna Briggs Institute’s guidance. The integrated data will then be synthesized using a thematic analysis approach to provide a comprehensive understanding of barriers and facilitators of international health care students’ well-being.

**Results:**

The initial literature search yielded 2408 papers from the selected databases. The findings of this review will be presented in a narrative format, supported by visualizations such as tables and diagrams. The review is expected to be completed by December 2024.

**Conclusions:**

This systematic integrative review will identify barriers and facilitators of international health care students’ well-being in higher education. The findings could inform the development of targeted interventions and support initiatives in higher education institutions globally, with the ultimate goal of enhancing the well-being as well as the academic and professional success of international health care students.

**Trial Registration:**

PROSPERO CRD42024372785; https://www.crd.york.ac.uk/prospero/display_record.php?ID=CRD42024372785

**International Registered Report Identifier (IRRID):**

PRR1-10.2196/59927

## Introduction

### Background

International health care students face unique compound stressors as they navigate the demands of health care education while adjusting to a new cultural environment [1]. Research indicates that health care students, for example, medical students, report significantly higher rates of depression, anxiety, and burnout compared to the general population [2]. As international students, these challenges are further compounded by the stress of addressing emerging challenges in the transition to culturally new environments [3,4]. Reports show that international students have significantly lower satisfaction with their student experiences, poorer global life satisfaction, less social support, higher rates of mental health issues and various addictions, and are less likely to seek help [5,6]. In addition, international students face persistent language barriers, cultural disparities, different health care systems, a lack of sense of belonging, and potential discrimination [7-10]. Thus, they are often allocated the identity of minority or vulnerable groups [10,11]. The enduring impacts of the COVID-19 pandemic, along with future crises and uncertainties, further threaten their overall well-being [12].

Moreover, poor well-being among international health care students can have profound consequences for patient care in professional settings. Poor well-being may increase the likelihood of burnout or mental health issues, which in turn can heighten the risk of medical errors [13]. This is possibly due to insufficient resilience, weak coping mechanisms during early career development, or a lack of adequate support. These issues can persist over time, leading to suboptimal patient care and professional dissatisfaction in the later stages of their careers [14]. Therefore, prioritizing the well-being of international health care students is crucial not only for their personal welfare but also for maintaining the quality and safety of health care services for patients [15].

The well-being of international health care students is a critical issue that requires comprehensive support systems and targeted interventions. Evidence suggests that some interventions have shown promise in reducing symptoms of depression, anxiety, and burnout among students, such as mindfulness-based programs or peer support programs [16,17]. These interventions not only enhance international health care students’ well-being during their academic journey but also equip them with resilience-building tools that benefit their future careers. Understanding barriers and facilitators of international health care students’ well-being could inform the development of effective interventions to promote their well-being. By understanding the unique challenges and needs of these students, this review will provide valuable insights that might support the next generation of health care students and in turn, professionals to develop sustainable resilience in their academic journey, future career, and ever-changing social environments. By investigating their barriers and facilitators of well-being, stakeholders will be able to identify areas for improvement within the educational and health care systems and develop culturally sensitive, inclusive interventions to address their unmet needs. Continued research in this field can provide insights for crafting tailored interventions and implementing systemic changes to bolster the well-being of upcoming health care professionals.

Numerous studies and systematic reviews have investigated the well-being of international students in higher education [18-20]. These studies predominantly address the stressors and psychological challenges faced by international students, especially during the COVID-19 pandemic [19]. Findings from these reviews consistently indicate a high prevalence of anxiety, depression, and stress among these student populations globally [18]. Factors such as self-esteem, emotional regulation, and social relationships that significantly influence student well-being [19] have been highlighted. School-based, well-being interventions have shown small to moderate positive effects on students’ social-emotional and behavioral adjustments [21]. However, these interventions may not fully address the specific needs and experiences of international students, particularly those in health care settings where stressors may differ.

The majority of reviews focus on specific populations, such as Chinese international students, or specific professions, such as nursing, thus limiting the generalizability of the findings [18]. For example, a scoping review identified common challenges of international nursing students in major host countries as language and communication barriers, cultural barriers, lack of familiarity with the health system, social exclusion, discrimination, financial struggles, and inability to maintain work-life balance [22]. The review also identified coping strategies including support from family and friends, peer support, seeking clarifications, maintenance of home countries’ cultural values, and development of language proficiency. There is a need for more reviews that encompass a broader range of international student experiences across different cultural backgrounds and health care disciplines [18,20]. Given the stressful nature of health care education, further exploration in this area is critical to develop targeted support mechanisms [23].

### Objective

This systematic integrative review aims to provide a comprehensive understanding of the barriers and facilitators of international health care students’ well-being in higher education.

### Review Question

What are the barriers and facilitators of international health care students’ well-being in higher education?

## Methods

### Study Design

To explore the well-being of international health care students, an integrative review approach was selected to address the research objective. This approach is particularly effective for synthesizing diverse types of research, including both experimental and nonexperimental studies [24]. The integrative review method was chosen for the following reasons:

- It is a flexible and iterative method that explores a complex phenomenon [25] from diverse sources with a more holistic approach [26].
- An integrative review is one of the more suitable approaches for identifying barriers and enablers [27,28] and evidence-based practice [24]. This aligns with the review question.

The overall design consulted the Joanna Briggs Institute’s guidance for a mixed methods systematic review, using a convergent integrated approach to synthesis and integration of the data [29].

### Protocol and Registration

The protocol was registered on May 1, 2024, with PROSPERO (CRD42024372785).

### Search Strategy

The search strings for this integrative review were developed in consultation with an experienced university librarian to enhance rigor and trustworthiness [24,30]. The search will use truncation and wildcard techniques to cover spelling variations and address information needs. A search log will be maintained for documentation purposes. Boolean operators will be used to combine the selected terms and formulate search strings based on the research question. The search strings are therefore based on search terms relating to international health care students, barriers and facilitators, and well-being. Search strings will be adjusted based on the specific characteristics of each database. This approach ensures that the search strategy aligns with the nuances and indexing systems of individual databases. We will conduct iterative running and testing before finalizing the search strings. In addition, further studies will be identified by a manual search of references and citation tracking of relevant studies using Google Scholar.

### Information Sources

Web of Science and Scopus are widely recognized databases spanning diverse scientific disciplines, frequently used for literature searches [31,32]. PubMed primarily focuses on biomedical and life sciences literature, making it an invaluable resource for health-related topics [33]. Moreover, EBSCOhost functions as an academic search engine, providing an interface to access numerous databases [34]. The databases searched through EBSCOhost in this review include Academic Search Complete; AgeLine; British Education Index; Business Source Complete; CINAHL Complete; eBook Collection (EBSCOhost); ERIC; European Views of the Americas: 1493 to 1750; GreenFILE; Historical Abstracts; Hospitality & Tourism Complete; Library, Information Science & Technology Abstracts; Psychology and Behavioral Sciences Collection; Regional Business News; SocINDEX with Full Text; Teacher Reference Center; UK & Ireland Reference Centre; MLA Directory of Periodicals; MLA International Bibliography; RILM Abstracts of Music Literature; APA PsycArticles; APA PsycInfo; APA PsycBooks; APA PsycExtra; and EconLit.

To supplement the review, a limited number of relevant and high-quality gray literature sources such as websites of the World Health Organization, United Nations, and relevant government agencies will be searched using a combination of terms relating to international health care students, barriers and facilitators, and well-being.

### Eligibility Criteria

To minimize the bias and ensure this systematic integrative review is transparent, predetermined inclusion and exclusion criteria will be applied based on Population, Phenomenon of Interest, and Context, as recommended in the Joanna Briggs Institute’s guidance for a mixed methods systematic review [29].

**Eligibility criteria.**

**Inclusion criteria**

- Population: (1) International health care students who move to another country to study in a cross-cultural context; (2) they are formally enrolled as full-time students in higher education institutions within the host countries; and (3) they undertake undergraduate or postgraduate academic health care programs, for example, master’s and PhD students
- Phenomenon of interest: Barriers and facilitators of international health care students’ well-being
- Context: The context for this review includes higher education institutions where international health care students pursue their studies at a third-level education including universities, colleges, medical schools, nursing schools, pharmacy schools, public health schools, and other relevant third-level educational institutions offering programs in health care–related fields
- Type of literature: (1) Qualitative studies such as interviews, focus groups, ethnographic studies, or qualitative surveys; (2) quantitative studies using surveys, questionnaires, or other quantitative methods; (3) mixed methods studies combining qualitative and quantitative approaches; and (4) peer-reviewed journal papers and gray literature including scientific or government reports, thesis, and conference papers
- Publication language: English
- Time period: No time restriction

**Exclusion criteria**

- Population: (1) International health care students studying part-time or through distance learning programs; (2) students enrolled in pre–health care or preparatory programs; and (3) health care students who work with animals
- Phenomenon of interest: Studies not relating to the well-being of international health care students
- Context: Postgraduate training solely based in health care institutions such as residency training or internship
- Type of literature: Case reports; editorials; opinion pieces; abstracts only; protocols; reviews; and posters
- Publication language: Non-English
- Time period: No time restriction

### Study Selection

All the studies from different databases will be uploaded into Covidence, which allows multiple reviewers to work simultaneously. Covidence will be used to remove duplicates and select eligible studies in 2 steps.

- Step 1 (title and abstract screening): Two reviewers (YX and JOD) will screen the study titles resulting from the finalized search strings. Irrelevant titles and abstracts will be excluded from further consideration.
- Step 2 (full-text screening): Two reviewers (YX and JOD) will conduct a full-text screening of studies selected from title and abstract screening based on the eligibility criteria already discussed.

Conflicts during the study selection will be resolved by mutual consensus. When consensus cannot be reached, a third reviewer (KPF) will be consulted.

### Quality Assessment

The Mixed Method Appraisal Tool (McGill University) will be used to appraise the quality of included studies [35]. This tool is chosen because it is suitable for appraising different types of primary studies including randomized controlled trials, observational studies, qualitative studies, and mixed method research. The outcome of the quality assessment will not be used to inform the inclusion of studies but will provide an overview of the methodological quality of the included papers.

### Data Extraction

The review will be a data extraction template in Covidence, developed based on the research question, to extract the following data items.

- Study characteristics: Author(s), type of study, year of publication, aim or purpose, study design, setting, sample size, and methods
- Participant characteristics: Demographics, country of origin, health care field of study, host country(s), student profile, gender, and target second language(s)
- Barriers: These refer to obstacles that hinder international health care students from achieving desired outcomes or accessing resources. This review focuses on identifying specific obstacles, difficulties, and limitations experienced by international health care students in pursuing their education in health care–related fields outside their home countries.
- Facilitators: These are elements that ease the path for international health care students to attain their goals such as supportive structures, enabling environments, interventions, services, programs, technologies, strategies, skills, or behaviors that lead to positive outcomes.
- Well-being indicators: According to the Organization for Economic Cooperation and Development, student well-being refers to the psychological, cognitive, social, and physical functioning and capabilities necessary for living a happy and fulfilling life [36].

This review adopts the Organization for Economic Cooperation and Development’s Programme for International Student Assessment indicators of well-being framework, examining 4 key dimensions of international health care students’ well-being: psychological, social, cognitive, and physical [37].

### Data Transformation and Integration

The extracted quantitative data will be transformed into qualitative data by providing narrative interpretations of the quantitative results to further describe the barriers and facilitators of international health care students’ well-being [29]. The transformed quantitative data (“qualitized” data) will then be integrated with the qualitative data to provide a comprehensive understanding of barriers and facilitators of international health care students’ well-being using a convergent integrated approach [29].

### Data Synthesis

The integrated data from the included studies will be synthesized using a thematic analysis method, which involves coding and categorizing data to identify common themes, patterns, and relationships [29]. A reflexive approach will be adopted for the thematic analysis [37]. This process will be carried out in 3 main stages: coding of primary data, development of descriptive themes, and generation of analytical themes [37]. After familiarizing with the data, coding will be done independently by 2 reviewers (YX and JOD) to identify distinct ideas (codes) in the integrated data using the highlight function of NVivo (Lumivero). Each group of similar codes will be aggregated, and a descriptive theme will be created to capture the meaning of each group by mutual agreement of the 2 reviewers (YX and JOD). Descriptive themes will be developed by comparing and contrasting the codes to identify areas of convergence, divergence, or complementarity. Analytical themes will be generated based on a third-order analytical interpretation of the descriptive themes by all the reviewers and this will go beyond the original data [37]. The generation of analytical themes will be iterative, involving regular discussion among the review team to refine interpretations and ensure coherence.

### Ethical Considerations

Ethical clearance is not deemed necessary for this integrative review, as it solely involves the synthesis of existing literature.

## Results

The review is expected to be finished by December 2024. Using the search strategy and databases discussed previously, an initial total of 2408 papers were identified, consisting of 427 papers from PubMed, 1125 papers from Scopus, 784 papers from Web of Science, and 72 papers from EBSCOhost (Multimedia Appendix 1). The synthesized findings will be presented thematically, supported by illustrative quotes, tables, or figures to enhance clarity and transparency. The study selection process will be depicted using the PRISMA (Preferred Reporting Items for Systematic Reviews and Meta-Analyses) flow diagram [38]. The review is expected to be completed by December 2024.

## Discussion

### Anticipated Findings

This systematic integrative review aims to contribute to a deeper understanding of the factors influencing the well-being of international health care students. The anticipated findings from the synthesis of data extracted from primary studies will include barriers and facilitators of international health care students’ well-being. It is expected that the barriers will be structural, systemic, environmental, or personal in nature [9,39,40]. On the other hand, facilitators will highlight factors that contribute to the well-being of international health care students in higher education such as supportive structures and interventions [41].

### Strengths and Limitations

This study, to the best of our knowledge, is the first systematic integrative review that focuses on barriers and facilitators of international health care students’ well-being in higher education. The integration of both qualitative and quantitative data is also an advantage as it will ensure a holistic and comprehensive coverage of factors influencing the well-being of international health care students [24-27].

Although a systematic integrative review is a rigorous approach, it does have inherent methodological shortcomings that can be challenging to overcome [25]. Combining diverse data sources can lead to complexity and potential bias, affecting the accuracy of the results [25]. To address potential bias, each stage of this review will be conducted by multiple independent reviewers to ensure a balanced assessment. In addition, the inclusion of only studies published in English could potentially lead to the exclusion of relevant studies conducted in other languages. However, we hope that English-language papers will provide sufficient depth and breadth as majority English-speaking countries such as the United States, United Kingdom, Canada, and Australia are the top host destinations of international students [22].

### Conclusions

The review will contribute to a deeper understanding of the challenges and support needs of international health care students. The findings may inform the development of appropriate interventions and support systems tailored to the specific needs of international health care students in a manner that supports their overall well-being.

## Appendix

Multimedia Appendix 1 Initial search result.

## Abbreviations

PRISMA: Preferred Reporting Items for Systematic Reviews and Meta-Analyses

## Data Availability

The datasets generated during and/or analyzed during this study are available from the corresponding author on reasonable request.
