# Supplementary material for: Barriers and Facilitators of International Health Care Students’ Well-Being in Higher Education: Protocol for a Systematic Integrative Review
Source: JMIR Res Protoc. 2024 Dec 11;13:e59927. doi: 10.2196/59927 (PMC11669885; doi:10.2196/59927)
Supplement: Multimedia Appendix 1 [file resprot_v13i1e59927_app1.docx]

**PubMed** (Limit to English)

| **Search number** | **Query** | **Results** |
| --- | --- | --- |
| **9** | #4 AND #7 AND #8 | 427 |
| **8** | well-being [Title/Abstract] OR wellbeing [Title/Abstract] OR "well being"[Title/Abstract] | 158,375 |
| **7** | #5 OR #6 | 10,892,245 |
| **6** | enabl*[Title/Abstract] OR support*[Title/Abstract] OR help*[Title/Abstract] OR facilitat*[Title/Abstract] OR assist*[Title/Abstract] OR advi*[Title/Abstract] OR opportunit*[Title/Abstract] OR support*[Title/Abstract] OR strateg*[Title/Abstract] OR intervention*[Title/Abstract] OR motivat*[Title/Abstract] | 7,075,297 |
| **5** | dilemma*[Title/Abstract] OR difficult*[Title/Abstract] OR limit*[Title/Abstract] OR hind*[Title/Abstract] OR hardship*[Title/Abstract] OR struggl*[Title/Abstract] OR problem*[Title/Abstract] OR issue*[Title/Abstract] OR fear*[Title/Abstract] OR experience*[Title/Abstract] OR hurdle*[Title/Abstract] OR plight*[Title/Abstract] OR demotivat*[Title/Abstract] OR adversity[Title/Abstract] | 5,932,466 |
| **4** | #1 AND #2 AND #3 | 9,066 |
| **3** | student*[Title/Abstract] | 385,347 |
| **2** | medic*[Title/Abstract] OR nursing*[Title/Abstract] OR nurse* [Title/Abstract] pharmac*[Title/Abstract] OR health*[Title/Abstract] OR dent*[Title/Abstract] OR healthcare*[Title/Abstract] OR clinic*[Title/Abstract] OR Physician**[Title/Abstract] OR Doctor*[Title/Abstract] | 9,136,294 |
| **1** | international [Title/Abstract] OR foreign [Title/Abstract] OR oversea*[Title/Abstract] OR migrant [Title/Abstract] OR alien[Title/Abstract] OR sojourn[Title/Abstract] OR Non-native*[Title/Abstract] | 571,910 |

**Scopus** (Limit to English)

| **Search number** | **Query** | **Results** |
| --- | --- | --- |
| **9** | #4 AND #7 AND #8 | 1125 |
| **8** | TITLE-ABS-KEY ( well-being OR wellbeing OR "well being" ) | 371,989 |
| **7** | #5 OR #6 | 30,827,587 |
| **6** | TITLE-ABS-KEY ( ( enabl* OR support* OR help* OR facilitat* OR assist* OR advi* OR opportunit* OR strateg* OR intervention* OR motivat* ) ) AND ( LIMIT-TO ( LANGUAGE , "English" ) ) | 18,986,035 |
| **5** | TITLE-ABS-KEY ( ( dilemma* OR difficult* OR limit* OR hind* OR hardship* OR struggl* OR problem* OR issue* OR fear* OR experience* OR hurdle* OR plight* OR demotivat* OR adversity ) ) AND ( LIMIT-TO ( LANGUAGE , "English" ) ) | 17,775,936 |
| **4** | #1 AND #2 AND #3 | 27,323 |
| **3** | TITLE-ABS-KEY ( student* ) | 1,558,189 |
| **2** | TITLE-ABS-KEY ( ( medic* OR nursing* OR nurse* OR pharmac* OR health* OR dent* OR healthcare* OR clinic* OR physician* OR doctor* ) ) AND ( LIMIT-TO ( LANGUAGE , "English" ) ) | 19,075,210 |
| **1** | TITLE-ABS-KEY ( ( international OR foreign OR oversea* OR migrant OR alien OR sojourn OR non-native* ) ) AND ( LIMIT-TO ( LANGUAGE , "English" ) ) | 2,501,920 |

**Web of Science** (Limit to English)

| **Search number** | **Query** | **Results** |
| --- | --- | --- |
| **9** | #4 AND #7 AND #8 | 784 |
| **8** | TS=( "well-being" OR wellbeing OR "well being") and English (Languages) | 243,933 |
| **7** | #5 OR #6 | 22,053,406 |
| **6** | TS=(enabl* OR support* OR help* OR facilitat* OR assist* OR advi* OR opportunit* OR strateg* OR intervention* OR motivat*) and English (Languages) | 13,209,639 |
| **5** | TS=(dilemma* OR difficult* OR limit* OR hind* OR hardship* OR struggl* OR problem* OR issue* OR fear* OR experience* OR hurdle* OR plight* OR demotivat* OR adversity) and English (Languages) | 13,132,467 |
| **4** | #1 AND #2 AND #3 | 15,360 |
| **3** | (TS=(student* )) AND (LA==("ENGLISH")) | 1,017,271 |
| **2** | (TS=(medic* OR nursing* OR nurse* OR pharmac* OR health* OR dent* OR healthcare* OR clinic* OR physician* OR doctor*)) AND (LA==("ENGLISH")) | 11,329,854 |
| **1** | (TS=(( international OR foreign OR oversea* OR migrant OR alien OR sojourn OR non-native* ) )) AND (LA==("ENGLISH")) | 1,438,207 |

**EBSCOhost** (Limit to English)

| **Search number** | **Query** | **Results** |
| --- | --- | --- |
| **10** | #4 AND #7 AND #8 (Exact duplicates removed from the results ) | 72 |
| **9** | #4 AND #7 AND #8 | 109 |
| **8** | TI ( "well-being" OR "mental health" OR wellbeing OR "well being") OR AB ( "well-being" OR "wellbeing" OR "well being") | 638,606 |
| **7** | #5 OR #6 | 3,544,518 |
| **6** | TI ( (enabl* OR support* OR help* OR facilitat* OR assist* OR advi* OR opportunit* OR strateg* OR intervention* OR motivat* ) OR AB ( (enabl* OR support* OR help* OR facilitat* OR assist* OR advi* OR opportunit* OR strateg* OR intervention* OR motivat* ) | 1,478,418 |
| **5** | TI ( dilemma* OR difficult* OR limit* OR hind* OR hardship* OR struggl* OR problem* OR issue* OR fear* OR experience* OR hurdle* OR plight* OR demotivat* OR adversity ) OR AB ( dilemma* OR difficult* OR limit* OR hind* OR hardship* OR struggl* OR problem* OR issue* OR fear* OR experience* OR hurdle* OR plight* OR demotivat* OR adversity ) | 2,121,081 |
| **4** | #1 AND #2 AND #3 | 9,450 |
| **3** | TI student* OR AB student* | 3,708,028 |
| **2** | TI ( medic* OR nursing* OR nurse* OR pharmac* OR health* OR dent* OR healthcare* OR clinic* OR physician* OR doctor ) AND AB ( medic* OR nursing* OR nurse* OR pharmac* OR health* OR dent* OR healthcare* OR clinic* OR physician* OR doctor ) | 3,620,874 |
| **1** | TI ( international OR foreign OR oversea* OR migrant OR alien OR sojourn OR non-native* ) OR AB ( international OR foreign OR oversea* OR migrant OR alien OR sojourn OR non-native* ) | 4,600,233 |

Database - Academic Search Complete;AgeLine;British Education Index;Business Source Complete;CINAHL Complete;eBook Collection (EBSCOhost);ERIC;European Views of the Americas: 1493 to 1750;GreenFILE;Historical Abstracts;Hospitality & Tourism Complete;Library, Information Science & Technology Abstracts;Psychology and Behavioral Sciences Collection;Regional Business News;SocINDEX with Full Text;Teacher Reference Center;UK & Ireland Reference Centre;MLA Directory of Periodicals;MLA International Bibliography;RILM Abstracts of Music Literature;APA PsycArticles;APA PsycInfo;APA PsycBooks;APA PsycExtra;EconLit
